# Multicentric Castleman Disease: A Case Report of an Arab Female

**DOI:** 10.7759/cureus.71112

**Published:** 2024-10-08

**Authors:** Mohamed K Mansour, Ahmed S Zayat

**Affiliations:** 1 Department of Hospital Medicine, Integrated Hospital Care Institute, Cleveland Clinic Abu Dhabi, Abu Dhabi, ARE; 2 Department of Internal Medicine, University Hospital Sharjah, Sharjah, ARE; 3 College of Medicine, University of Sharjah, Sharjah, ARE

**Keywords:** castleman disease, interleukin-6, lymphadenopathy, sjögren syndrome, tocilizumab

## Abstract

We report the case of a 74-year-old female, who presented with a two-month history of fever, night sweats, and lymphadenopathy. She was thoroughly investigated, with high clinical suspicion for lymphoma. However, lymph node biopsy results revealed histopathological features of a hyaline vascular variant of Castleman disease. Owing to the involvement of multiple lymph node regions and the presence of systemic symptoms, the patient was diagnosed with multicentric Castleman disease and was started on steroid therapy, as well as anti-interleukin-6 (anti-IL-6) therapy, namely, tocilizumab, for the management of this condition. The patient responded well to the treatment and was discharged home in stable condition.

## Introduction

Castleman disease is a rare disease that was first described by Benjamin Castleman in the 1950s, as a benign group of enlarged lymph nodes found in the mediastinum of asymptomatic patients [1]. It represents a group of lymphoproliferative disorders that is characterized by a spectrum of histopathological features [1]. These histopathological features take the form of nonneoplastic lymph node hypertrophy and angiofollicular lymph node hyperplasia [1,2]. Castleman disease is clinically subclassified into unicentric Castleman disease and multicentric Castleman disease [1]. Unicentric Castleman disease is usually asymptomatic and involves a single lymph node region; on the other hand, multicentric Castleman disease causes systemic manifestations and involves multiple lymph node regions [3,4]. There is limited data on the epidemiology of Castleman disease, and its etiology is not well understood. Human herpes virus-6 (HHV-6) is thought to play a role in the pathogenesis of the disease, in addition to immune dysregulation marked by elevated serum levels of interleukin-6 (IL-6), which is responsible for the systemic manifestations in multicentric Castleman disease [4,5]. There are limited randomized clinical trials on Castleman disease therapy, and most case reports and case series reported response to debulking surgery, high doses of corticosteroids, adjuvant chemotherapy, and rituximab or IL-6 inhibitors [6-9]. It is not clear yet if using a high or a low dose of the IL-6 inhibitor tocilizumab would make a difference, what would be the long-term outcome after 12 months of therapy, and how should the treatment be tapered or stopped.

## Case presentation

**Figure 1:**
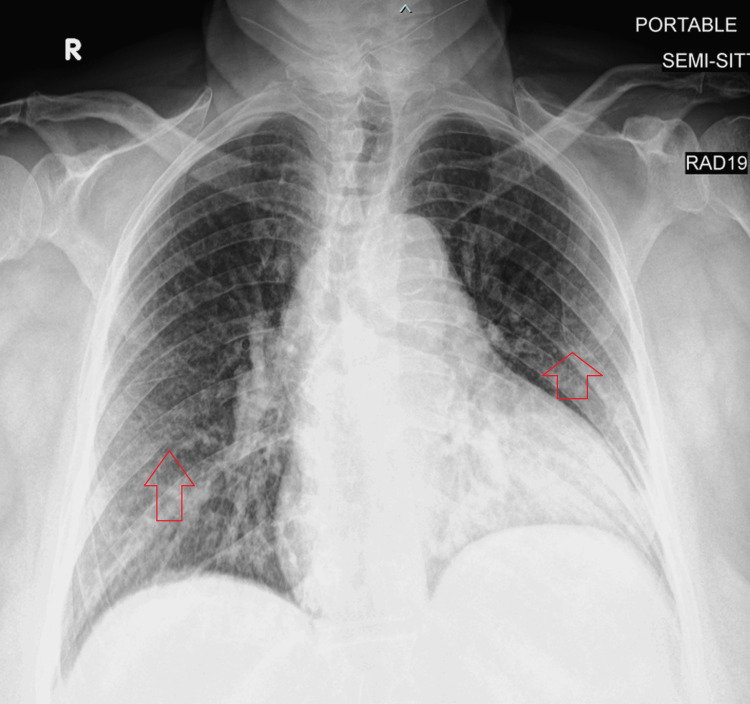
Portable anteroposterior chest X-ray showing bilateral interstitial changes with no consolidation

We present the case of a 74-year-old female, of Arabic ethnicity, who has a background history of hypertension, type 2 diabetes mellitus, dyslipidemia, hypothyroidism, and Sjögren syndrome and who presented to the hospital with a history of fever of two-month duration. The patient reported fatigue and night sweats for the same duration of time. She also complained of a dry nonproductive cough. There was no history of skin rash, joint pain, or Raynaud's phenomenon. The review of systems was otherwise negative. The patient was clinically diagnosed with Sjögren syndrome based on a six-year history of oral and ocular sicca with antinuclear antibody (ANA) positivity (1:1,000 granular pattern), high IgG of 3,408 mg/dL (reference range: 64-422), and a positive Schirmer's test. However, she had negative Sjögren syndrome antigen A (SSA) and Sjögren syndrome antigen B (SSB) antibodies and low positive polymyositis-scleroderma 75 (PM-SCL 75) antibodies. IgG subclasses did not show any IgG4 predominance with IgG2, IgG3, and IgG4 of 427 (reference range: 130-555), 208 (reference range: 15-102), and 232 mg/dL (reference range: 2-96), respectively. Her blood electrophoresis showed a normal lambda-to-kappa ratio. She never had a labial gland biopsy to confirm the clinical diagnosis, and she was treated with hydroxychloroquine 200 mg daily and pilocarpine 5 mg twice daily. The patient's other home medications included amlodipine, metformin, atorvastatin, and levothyroxine. On examination, her vital signs were as follows: temperature of 38.3°C, pulse rate of 78 beats per minute, respiratory rate of 19 breaths per minute, blood pressure of 124/65 mmHg, and oxygen saturation of 98% on room air. She had evidence of bilateral cervical and axillary lymphadenopathy with the largest lymph node measuring 2×2 cm in the left axilla, but there was no evidence of hepatosplenomegaly. Initial laboratory investigations revealed serum hemoglobin of 7.9 g/dL (normal: 11.5-15.5), white cell count of 21,500 cells/mcL (normal: 3,500-10,000) with a neutrophil count of 15,000 cells/mcL and lymphocyte count of 4,200 cells/mcL, normal liver function tests, C-reactive protein of 131 mg/L (normal: 0-10), estimated sedimentation rate of 140 mm/hour (normal: 0-14), and procalcitonin of 0.04 ng/mL (normal: <0.15). Chest X-ray showed bilateral interstitial changes with no consolidation as shown in Figure 1.

**Figure 2:**
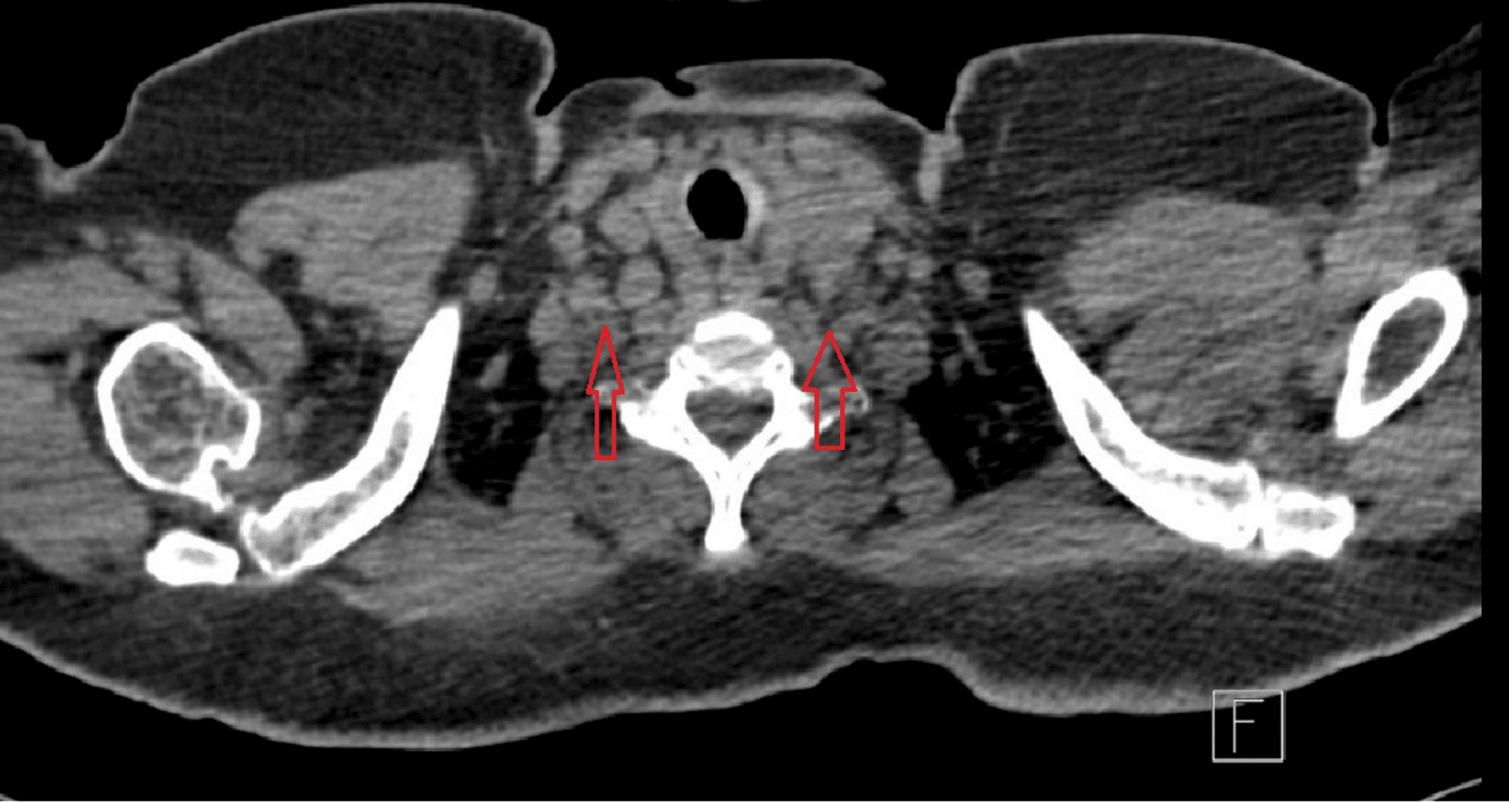
Lower neck cuts of a high-resolution CT scan of the chest showing multiple enlarged lymph nodes bilaterally

**Figure 3:**
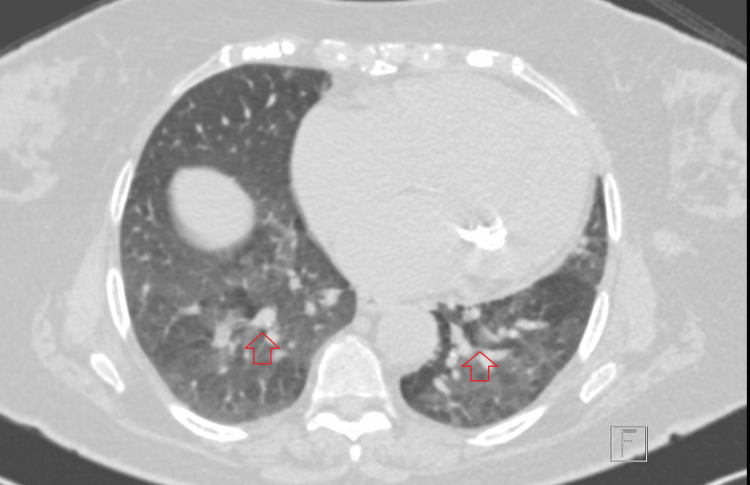
High-resolution CT scan of the chest showing mild interstitial changes and no consolidation

A high-resolution CT scan of the chest was done for further assessment and showed multiple enlarged lymph nodes in the lower jugular, subclavian, and axillary chains bilaterally, in addition to mild interstitial changes and no consolidation as shown in Figure 2 and Figure 3.

**Figure 4:**
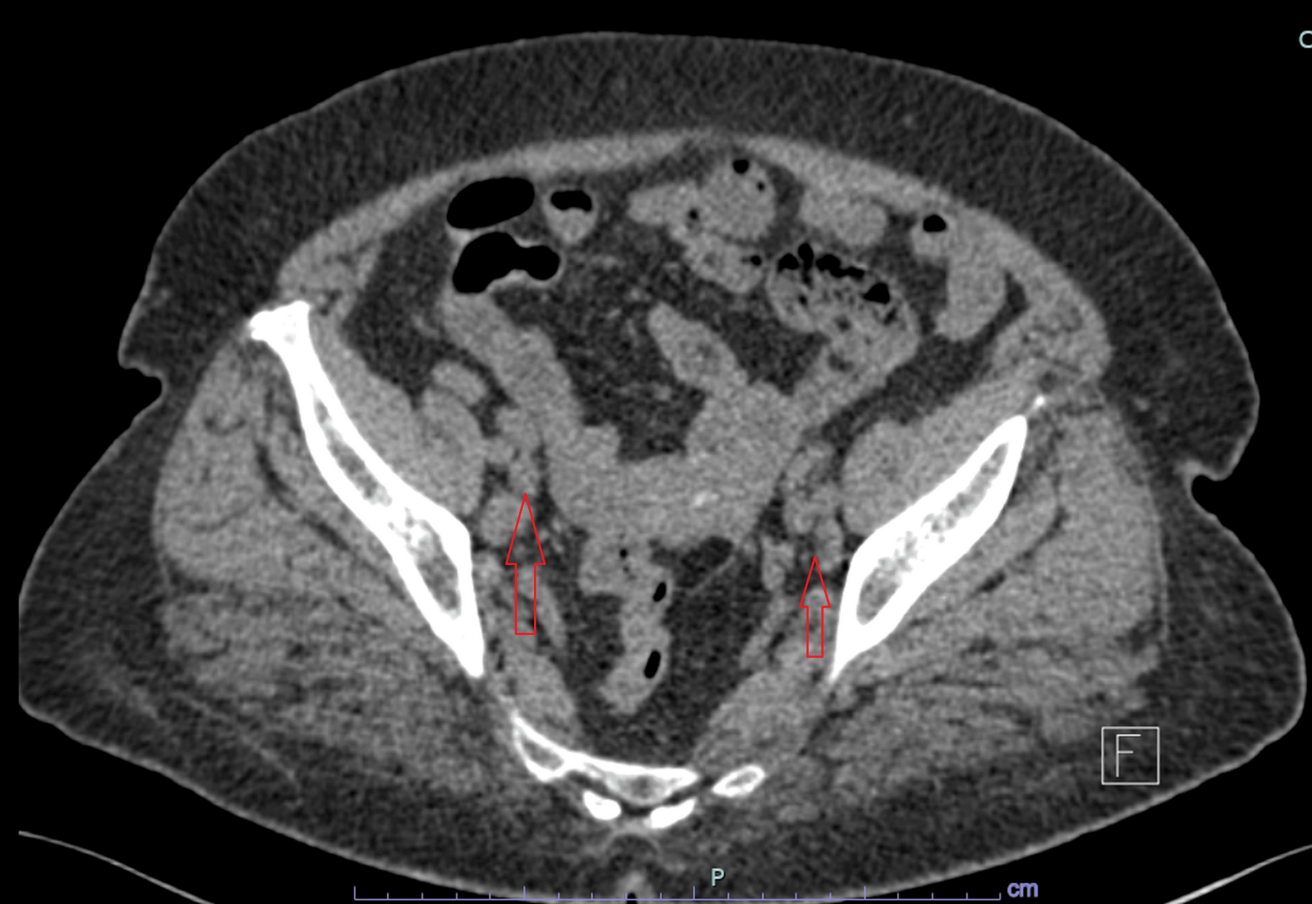
CT scan of the pelvis showing multiple enlarged lymph nodes

A CT scan of the abdomen and pelvis showed multiple enlarged intra-abdominal lymph nodes as shown in Figure 4; moderate hepatomegaly with fatty infiltration; normal-sized spleen, pancreas, and kidneys with no focal lesions; normal bowel loops with no wall thickening; normal uterus and adnexal structures; and no evidence of ascites or intra-abdominal fluid collections.

The patient was admitted to the hospital under the impression of community-acquired pneumonia and was started on broad-spectrum antibiotics. Blood culture and urine culture results showed no growth. The patient completed seven days of antibiotic therapy: piperacillin+tazobactam with no improvement in fever or inflammatory markers. Additional laboratory tests were ordered, including interleukin-6 (IL-6) levels, the results of which are outlined in Table 1. Human herpes virus-8 (HHV-8) testing was not done. Serum protein electrophoresis revealed a polyclonal rise in total globulin levels with no monoclonal spike (M-spike).

**Table 1:** Summary of the results of the laboratory tests performed on the patient PCR: polymerase chain reaction

Laboratory test	Value	Reference range
Hemoglobin (g/dL)	7.9	11.5-15.5
White cell count (cells/mcL)	21,500	3,500-10,000
C-reactive protein (mg/L)	131	0-10
Estimated sedimentation rate (mm/hour)	140	0-14
Procalcitonin (ng/mL)	0.04	<0.15
Lactate dehydrogenase (U/L)	138	120-246
Beta-2 microglobulin (mg/L)	2.9	0.4-2.6
Ferritin (ng/mL)	369	10-291
Serum calcium (mmol/L)	2.16	2.08-2.60
Angiotensin-converting enzyme level (U/L)	25	14-82
Interleukin-6 (pg/mL)	23	0-13
QuantiFERON	Negative	Negative
Epstein-Barr virus (EBV) PCR	Negative	Negative
Cytomegalovirus (CMV) PCR	Negative	Negative
Hepatitis B surface antigen	Negative	Negative
Anti-hepatitis C virus antibody	Negative	Negative
HIV-1 and HIV-2 antibody/antigen	Negative	Negative
Antinuclear antibody (ANA)	Positive	Negative
Anti-cyclic citrullinated peptide (anti-CCP)	Negative	Negative
Coronavirus disease 2019 (COVID-19)	Negative	Negative

**Figure 5:**
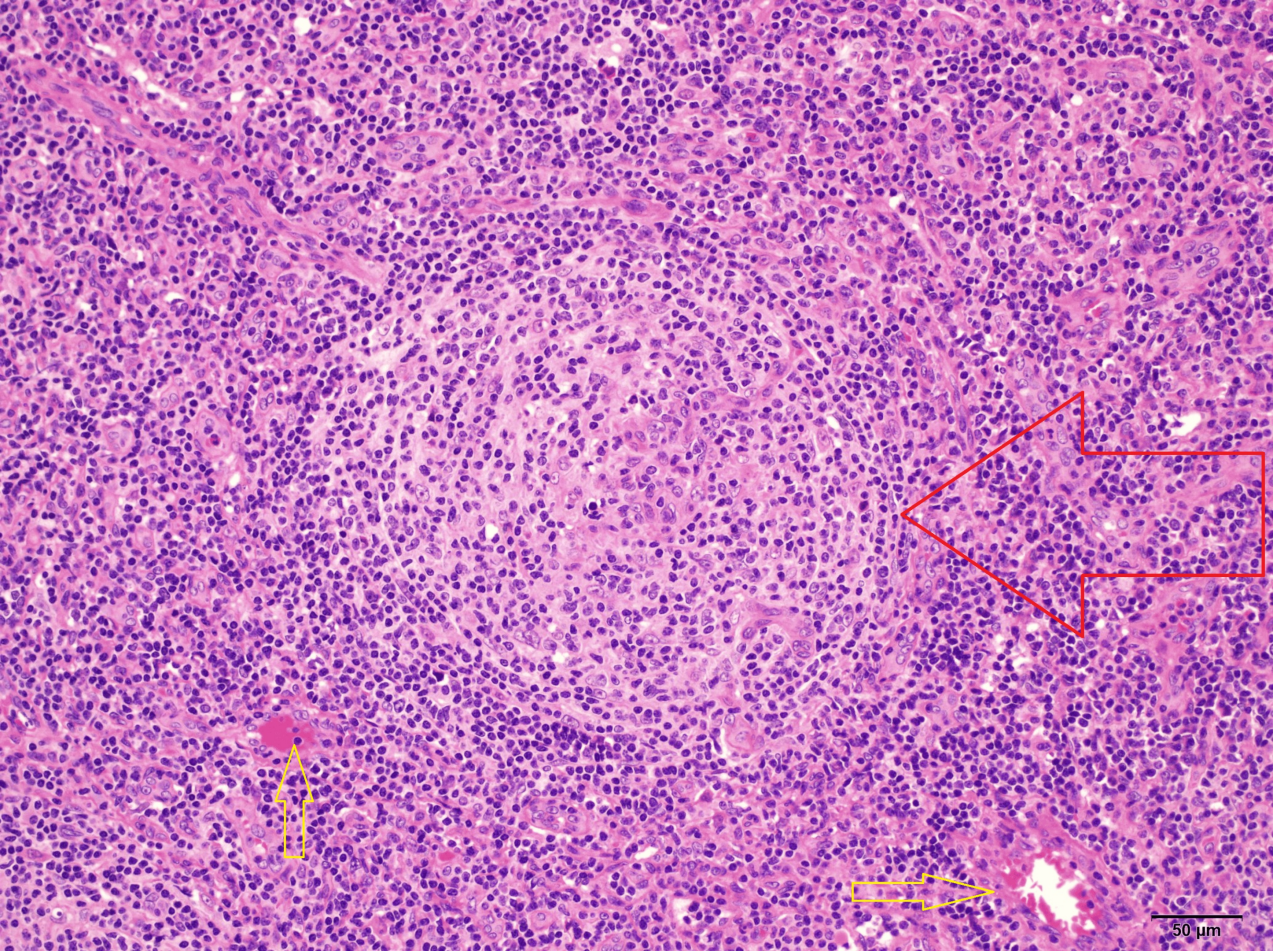
Lymphoid follicles with progressively transformed germinal centers Onion skin appearance (red arrow) with penetrating hyalinized blood vessels (yellow arrows)

An excisional biopsy of one of the axillary lymph nodes was performed, and histopathological evaluation revealed the presence of multiple small partially regressively transformed germinal centers surrounded by expanded mantle zones. There was no necrosis or granuloma formation. The onion ring pattern of mantle cells and the lollipop-like appearance of focal penetrating hyalinized arterioles were present, which are features consistent with the hyaline vascular variant of Castleman disease as shown in Figure 5. There were no immunomorphological features of lymphoma on the biopsy result.

**Figure 6:**
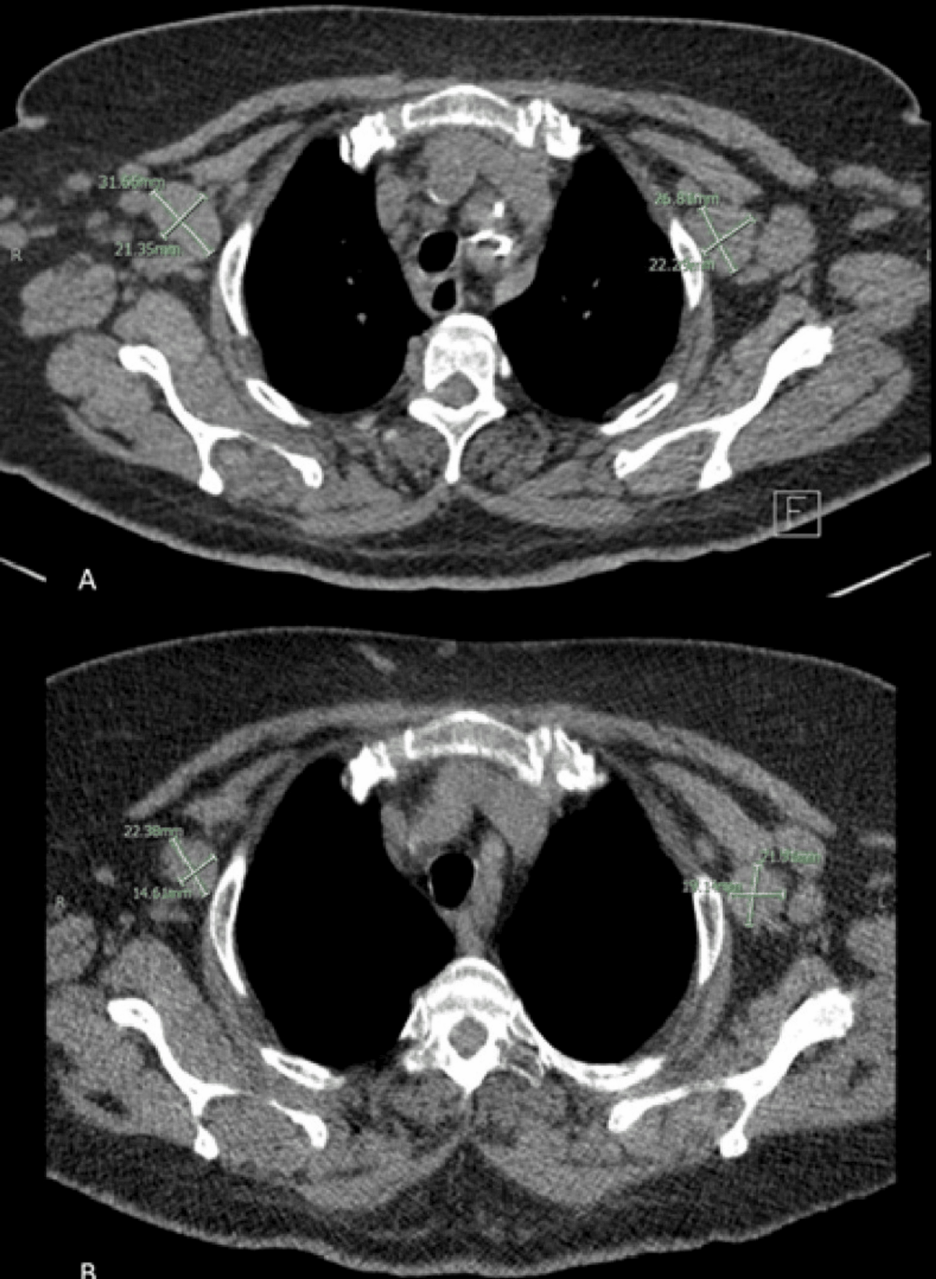
High-resolution CT scan of the chest showing multiple enlarged lymph nodes in the neck (a) before tocilizumab and (b) decrease in size after treatment with tocilizumab

The rheumatology team was consulted on the case, and the patient was subsequently started on 20 mg of prednisolone daily. In view of the raised serum IL-6 levels of 37 pg/mL, a decision was taken to start the patient on intravenous tocilizumab 4 mg/kg every four weeks. The patient was discharged home with a follow-up appointment at the rheumatology clinic. Three months later, the dose of tocilizumab was increased to 8 mg/kg every four weeks as the inflammatory markers and serum IL-6 levels continued to be raised. After two months of dose escalation, the patient had a complete resolution of fever and the absence of lymphadenopathy on clinical examination, and her inflammatory markers became normal. At that time, a high-resolution CT scan of the chest was done, which showed persistent lymphadenopathy; however, there was a regression in the size and number of the enlarged lymph nodes as shown in Figure 6. The dose of tocilizumab was further escalated to 8 mg/kg every two weeks, as IL-6 levels remained elevated. The patient continued to be symptom-free seven months later. She remains in good health and continues to follow up regularly in the rheumatology clinic with a plan to gradually taper the tocilizumab dose.

## Discussion

Castleman disease represents a group of lymphoproliferative disorders that share a spectrum of histopathological features [1]. It is characterized by nonneoplastic lymph node hypertrophy and histologically by angiofollicular lymph node hyperplasia [1]. It was first described in the 1950s by Benjamin Castleman as a benign group of enlarged lymph nodes found in the mediastinum of asymptomatic patients [2].

There are two main forms of Castleman disease clinically, namely, unicentric Castleman disease and multicentric Castleman disease [1]. Unicentric Castleman disease involves a single lymph node region, and it is usually asymptomatic, whereas multicentric Castleman disease involves multiple lymph node regions, and it is often associated with systemic manifestations [3,4].

Multicentric Castleman disease is further subdivided into three types, which include idiopathic multicentric Castleman disease (iMCD), human herpes virus-8-associated multicentric Castleman disease, and polyneuropathy, organomegaly, endocrinopathy, monoclonal plasma cell disorder, skin changes (POEMS)-associated Castleman disease [1,4,5]. Idiopathic multicentric Castleman disease is further subclassified into idiopathic multicentric Castleman disease-thrombocytopenia, ascites, reticulin fibrosis, renal dysfunction, and organomegaly (iMCD-TAFRO) or idiopathic multicentric Castleman disease-not otherwise specified (iMCD-NOS) [1].

Histologically, Castleman disease is classified into three types: hyaline vascular, plasma cell variant, and mixed variant Castleman disease. The hyaline vascular subtype is characterized by an "onion skin" pattern formed by lymphoid follicular hyperplasia that surrounds a hyalinized blood vessel. The plasma cell variant consists of many mature plasma cells surrounding large germinal centers. The mixed variant of the disease shares features common to the other two histologic subtypes [6].

Data on the epidemiology of Castleman disease is limited. Unicentric Castleman disease is diagnosed at a younger age than multicentric Castleman disease. There is no gender preference for unicentric Castleman disease; however, multicentric Castleman disease is more common in males [1]. Castleman disease can mimic a number of conditions such as lymphoma, acute infections particularly acute HIV infection, and autoimmune diseases such as systemic lupus erythematosus and rheumatoid arthritis, which should be considered in the differential diagnosis of the disease [1].

The etiology of Castleman disease is not well understood. Infection by human herpes virus-8 (HHV-8) is thought to play a role in the pathogenesis of the disease, in addition to immune dysregulation marked by elevated serum levels of interleukin-6 (IL-6), which is responsible for the systemic manifestations in multicentric Castleman disease [4,5].

We reported a case of histologically confirmed multicentric Castleman disease in a female of Arabic ethnicity, with a background diagnosis of Sjögren syndrome, who presented with pyrexia and lymphadenopathy and who responded clinically to tocilizumab 4 mg/kg every four weeks but failed to have complete resolution in lymphadenopathy and IL-6 levels even with escalated dose to 8 mg/kg every two weeks. Although there are several case reports, case series, and limited clinical trials on Castleman disease, most reported cases were from the Far East or Caucasian ethnicities [5,7-12], while this is one of the first few case reports of Castleman disease reported in a patient of Arabic ethnicity [13]. Although it is important to distinguish idiopathic multicentric Castleman disease from those cases that are associated with the human herpes virus-8 (HHV-8-associated multicentric Castleman disease), in terms of selecting the right therapeutic options, we could not test for HHV-8 in our case as testing for it was not available at our facility.

There have been several case reports of unicentric Castleman disease described in the literature. However, only a few cases of multicentric Castleman disease have been reported. Menezes et al. reported a case of a 71-year-old male who presented with a several-month history of constitutional symptoms and generalized lymphadenopathy and was eventually diagnosed with hyaline vascular multicentric Castleman disease after a lymph node biopsy. He was treated with systemic corticosteroid therapy, which provided symptomatic relief and resulted in disease remission [5].

In a case series of 16 patients with Castleman disease by Bowne et al., only three patients were diagnosed with multicentric disease. Two of these patients had a hyaline vascular variant of the disease histologically and achieved complete remission after adjuvant therapy using high-dose corticosteroids alone or in combination with suramin. The remaining patient had histologic features of the plasma cell variant of the disease. He was treated with surgery and systemic therapy using antineoplastic agents (vinblastine, cyclophosphamide, vincristine, and carmustine) but unfortunately died four months after presentation [7].

In another case series of 21 patients with Castleman disease by Chronowski et al., nine patients had multicentric Castleman disease. These patients were treated with combination chemotherapy (seven patients were treated with cyclophosphamide, vincristine, doxorubicin, and corticosteroids, while two patients were treated with chlorambucil and corticosteroids). Five of these patients demonstrated complete response and had no evidence of the disease, while the remaining four patients had progressive disease at the last follow-up visits [8].

In eight cases of multicentric Castleman disease reported by Herrada et al., three patients received combination chemotherapy that included cyclophosphamide, vincristine, doxorubicin, corticosteroids, and in one case chlorambucil and corticosteroids, which resulted in complete remission; two patients were treated with corticosteroids and required intermittent maintenance therapy with corticosteroids for disease reactivation; two patients were treated with surgical excision but unfortunately died, while the last patient received no specific treatment and had spontaneous regression of symptoms [9].

The coexistence of Castleman disease and Sjögren syndrome has been described previously in the literature [10,11]. In one case report of a patient with multicentric Castleman disease and Sjögren syndrome, biological therapy using the anti-cluster of differentiation 20 (CD20) monoclonal antibody rituximab resulted in clinical remission [10]. In another case report of a patient with multicentric Castleman disease; thrombocytopenia, ascites, reticulin fibrosis, renal dysfunction, and organomegaly (TAFRO) syndrome; and Sjögren syndrome, there was a poor response to anti-CD20 therapy with rituximab and anti-IL-6 therapy with siltuximab [11]. In a third case report by Pan et al. of a patient with Castleman disease, Sjögren syndrome, and secondary membranous nephropathy, the patient developed clinical remission after therapy with tocilizumab, cyclophosphamide, and methylprednisolone [12].

Lymphocytes and plasma cells are the predominant cells infiltrating the lymph nodes of patients with Castleman disease and the salivary glands of patients with Sjögren syndrome [12]. IL-6 produced by the local B-cells is important for the growth, differentiation, and survival of lymphocytes and plasma cells, resulting in lymph node hyperplasia and hypergammaglobulinemia [12]. It can also induce vascular endothelial growth factor (VEGF) secretion and increase vascular permeability [12]. This can explain many of the disease symptoms such as lymphadenopathy, edema, pleural effusions, and ascites, which patients tend to experience. IL-6 dysregulates the humoral immune response resulting in positive ANA in approximately one-third of the patients [14]. This could be the reason for the previous diagnosis of Sjögren disease in our case, and her sicca symptomology could be a feature of the prodromal presentation of multicentric Castleman disease.

Several treatments have been used for the management of multicentric Castleman disease, particularly the idiopathic type, namely, surgery, corticosteroids, rituximab, or chemotherapy [12]. Monoclonal antibodies targeting the interleukin-6 signaling pathway such as tocilizumab have been approved and are currently being used for the management of this disease condition [12]. The safety and efficacy of tocilizumab were first reported by Nishimoto et al., where all patients responded to 8 mg/kg two weekly dosing for 16 weeks, followed by continued dosing at the discretion of the investigator, mostly at two weekly intervals (53% of the patients) or four weekly intervals (18% of the patients). The patients showed a significant reduction in lymphadenopathy by 16 weeks, with 52% of the patients achieving lymph node sizes of less than 1 cm by one year [15].

In our reported case, IL-6 levels remained elevated after tocilizumab treatment and did not respond to changes in therapy. However, despite that, she experienced an improvement in her symptoms and a reduction in the size and number of enlarged lymph nodes, after therapy with tocilizumab.

## Conclusions

This case report emphasizes the importance of histopathological diagnosis in patients presenting with lymphadenopathy and systemic symptoms. It also reminds the clinician to consider Castleman disease in the differential diagnosis of patients presenting with such symptoms. Lastly, it adds to the limited available literature on multicentric Castleman disease.
